# A telomere-to-telomere reference genome of ficus (*Ficus hispida*) provides new insights into sex determination

**DOI:** 10.1093/hr/uhad257

**Published:** 2023-12-13

**Authors:** Zhenyang Liao, Tianwen Zhang, Wenlong Lei, Yibin Wang, Jiaxin Yu, Yinghao Wang, Kun Chai, Gang Wang, Huahao Zhang, Xingtan Zhang

**Affiliations:** Shenzhen Branch, Guangdong Laboratory for Lingnan Modern Agriculture, Genome Analysis Laboratory of the Ministry of Agriculture, Agricultural Genomics Institute at Shenzhen, Chinese Academy of Agricultural Sciences, Shenzhen, Guangdong 518120, China; CAS Key Laboratory of Tropical Forest Ecology, Xishuangbanna Tropical Botanical Garden, Chinese Academy of Sciences, Mengla, Yunnan 666303, China; College of Landscape Architecture, Fujian Agriculture and Forestry University, Fuzhou 350002, China; Shenzhen Branch, Guangdong Laboratory for Lingnan Modern Agriculture, Genome Analysis Laboratory of the Ministry of Agriculture, Agricultural Genomics Institute at Shenzhen, Chinese Academy of Agricultural Sciences, Shenzhen, Guangdong 518120, China; Shenzhen Branch, Guangdong Laboratory for Lingnan Modern Agriculture, Genome Analysis Laboratory of the Ministry of Agriculture, Agricultural Genomics Institute at Shenzhen, Chinese Academy of Agricultural Sciences, Shenzhen, Guangdong 518120, China; Shenzhen Branch, Guangdong Laboratory for Lingnan Modern Agriculture, Genome Analysis Laboratory of the Ministry of Agriculture, Agricultural Genomics Institute at Shenzhen, Chinese Academy of Agricultural Sciences, Shenzhen, Guangdong 518120, China; Shenzhen Branch, Guangdong Laboratory for Lingnan Modern Agriculture, Genome Analysis Laboratory of the Ministry of Agriculture, Agricultural Genomics Institute at Shenzhen, Chinese Academy of Agricultural Sciences, Shenzhen, Guangdong 518120, China; Shenzhen Branch, Guangdong Laboratory for Lingnan Modern Agriculture, Genome Analysis Laboratory of the Ministry of Agriculture, Agricultural Genomics Institute at Shenzhen, Chinese Academy of Agricultural Sciences, Shenzhen, Guangdong 518120, China; CAS Key Laboratory of Tropical Forest Ecology, Xishuangbanna Tropical Botanical Garden, Chinese Academy of Sciences, Mengla, Yunnan 666303, China; College of Pharmacy and Life Science, Jiujiang University, Jiujiang 332005, China; Shenzhen Branch, Guangdong Laboratory for Lingnan Modern Agriculture, Genome Analysis Laboratory of the Ministry of Agriculture, Agricultural Genomics Institute at Shenzhen, Chinese Academy of Agricultural Sciences, Shenzhen, Guangdong 518120, China

## Abstract

A high-quality reference genome is indispensable for resolving biologically essential traits. *Ficus hispida* is a dioecious plant. A complete *Ficus* reference genome will be crucial for understanding their sex evolution and important biological characteristics, such as aerial roots, mutualistic symbiosis with ficus-wasps, and fruiting from old stems. Here, we generated a telomere-to-telomere (T2T) genome for *F. hispida* using PacBio HiFi and Oxford Nanopore Ultra-long sequencing technologies. The genome contiguity and completeness has shown improvement compared with the previously released genome, with the annotation of six centromeres and 28 telomeres. We have refined our previously reported 2-Mb male-specific region into a 7.2-Mb genomic region containing 51 newly predicted genes and candidate sex-determination genes *AG2* and *AG3*. Many of these genes showed extremely low expression, likely attributed to hypermethylation in the gene body and promoter regions. Gene regulatory networks (GRNs) revealed that *AG2* and *AG3* are related to the regulation of stamen development in male flowers, while the *AG1* gene is responsible for regulating female flowers’ defense responses and secondary metabolite processes. Comparative analysis of GRNs showed that the NAC, WRKY, and MYB transcription factor families dominate the female GRN, whereas the MADS and MYB transcription factor families are prevalent in the male GRN.

## Introduction

Recently, studies on sex determination in some plant species have advanced understanding of the genetic basis of sex determination and provided essential information for breeding, such as grapes [1]. *Ficus hispida*, a functionally dioecious plant, is so far unstudied. Although a reference genome has been released [2], there are still numerous gaps.

The genus *Ficus* L. (family Moraceae) is one of the largest angiosperm genera*,* with over 800 species of moderate woody plants, epiphytes, and shrubs [3] predominantly distributed in the tropical and subtropical regions of Asia, Oceania, Africa, and the Americas. *Ficus* species serve as a valuable food source and habitat for numerous organisms and play a crucial role in tropical rainforest ecosystems [4–6]. The syconium inflorescence, also known as the ficus fruit, is vital in the interaction between the ficus trees and their specialist pollinating wasps [7]. Typically, each species of ficus tree relies on a specialist pollinator wasp to enter the fruit cavity through the mouth of the bracts and pollinate the female flowers inside. This interaction establishes a mutually beneficial relationship between the wasp and the ficus tree. The fruit serves as a food source for the wasps and offers an environment to reproduce their offspring, resulting in a close and highly specialized mutualistic symbiosis [7, 8].

In the genus *Ficus* functional dioecy evolved from monoecy [2, 9, 10]. The flowers primarily consist of three types: female, male, and gall. When the pollinating wasp lays eggs inside a female flower’s ovary, specific tissues proliferate asexually, causing ovaries to enlarge and form gall flowers. Wasps use female flowers in monoecious plants to lay eggs for parasitism and reproduction. In contrast, in dioecious trees, female fruits contain only female flowers, and male ones contain both male and gall flowers [11]. The ovipositor of the pollinating fig wasp can only reach the ovary to form gall flowers through the short-style female flowers. Hence, the length of the style determines the final fate of the ficus female flowers, which may be a crucial trait for the functional differentiation of the sexes [12]. Flower development is mainly categorized into four stages, which differ in species with different relationships between *Ficus* and wasps [13]. Stage A represents the period before pollinator-ready female or egg-laying gall flowers mature. At stage B, the bracts of the fruit open, specific volatiles are emitted to attract wasps into the fruit cavity [14], and the female flowers become receptive to pollination, while the gall flowers are prepared for egg-laying. In the C period, the pollinated female flower’s ovaries continue to develop, while the gall flowers undergo expansion to accommodate egg-laying. Period D, known as the staminate stage, marks the maturation of male flowers. During this stage, the anthers dehisce and release pollen. After mating, the female wasps depart from the ficus fruit and carry the pollen. The ficus fruit wall softens and pulpifies during the late flowering period while the seeds mature [14].

**Figure 1:**
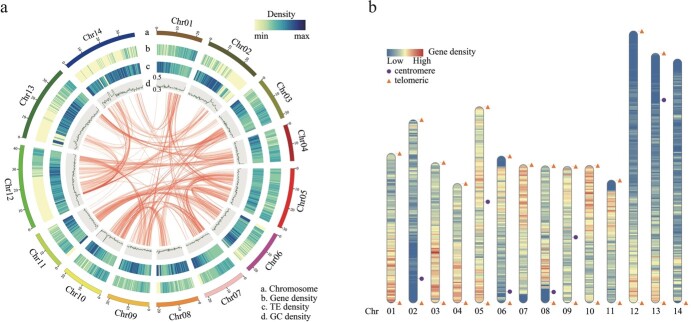
T2T genome assembly of *F. hispida*. **a** Circle diagram of basic genome information. Concentric circles from outside to inside represent chromosomes, gene density, repeat density, and GC density. Lines in the interior indicate collinearity. **b** Telomere and centromere detection map. Orange triangles and purple circles represent telomeres and centromere within the assembled genome; blue indicates low gene density; red indicates high gene density.

The genus *Ficus* contains two monoecious subgenera (*Urostigma* and *Pharmacosycea*) and four functionally dioecious subgenera (*Sycomorus*, *Ficus*, *Synoecia*, and *Sycidium*) [9]. Sex determination in *Ficus* adheres to the classical theory of sex chromosome evolution, where dioecy emerged from monoecy, and this transition occurred ~47.5 million years ago [2, 9, 10]. *Ficus hispida* is a dioecious species of the subgenus *Sycomorus* [15]. The *F. hispida* (2*n* = 2*x* = 28) genome size is 360 Mb [2], and its phased X and Y sex chromosomes were assembled separately [2]. A recently evolved sex-determination region spanning ~2 Mb on chromosome Y was identified [2]*,* including the candidate sex-determination gene *FhAG2* (a MADS-box transcription factor). Two duplicates, *FhAG1* and *FhAG3*, were found elsewhere in the genome [2]. However, there are many gaps in the assembled sex chromosomes due to the presence of highly repeated sequences.

We describe a telomere-to-telomere (T2T) gap-free genome assembly combining PacBio with nanopore sequencing data. Furthermore, we re-analyzed the male-specific region using the resequencing data. Genome-wide methylation levels were assessed based on nanopore data. We constructed gene regulatory networks for ficus fruit development in males and females.

## Results

### A telomere-to-telomere genome for *Ficus* species

We sequenced male individuals of *F. hispida*, which possess X and Y chromosomes [2]. The genome was estimated to be 366 Mb based on Illumina short reads [2]. Different sequencing platforms were employed to construct a high-quality T2T genome. We obtained 35.2 Gb of clean HiFi reads based on the PacBio Sequel II platform, with an approximate read N50 of 22.5 kb and a sequencing depth of 100× (Supplementary Data Table S1). Additionally, we generated 45.7-Gb ultra-long reads using Oxford Nanopore Technology (ONT), with the read N50 and sequencing coverage around 71.0 kb and 120×, respectively (Supplementary Data Table S1).

For genome assembly, we used different assembly strategies. We generated a preliminary genome assembly using high-quality HiFi reads, producing 62 contigs with the N50 size of 24.3 Mb (Supplementary Data Table S2). *De novo* assembly was performed using ultra-long reads, resulting in a contig-level assembly with an N50 of 21.2 Mb and containing 47 contigs (Supplementary Data Table S2). The ONT-based assembly genome was utilized to fill the gaps in the HiFi-based assembly, excluding redundant sequences and short contigs. Finally, a gap-free genome of 371.8 Mb was constructed, with 14 large contigs representing 14 chromosomes, with an N50 length of 23.1 Mb (Supplementary Data Table S2). The basic structural features (gene density, repeat sequence density, and GC density) of the T2T genome are shown in Fig. 1a. The sequence shows strong collinearity with our previously released genome [2] (Supplementary Data Fig. S1).

We used the seven-base telomere sequence (CCCTAAA at the 5′ end or TTTAGGG at the 3′ end) as a query sequence to scan the genome. We identified 28 telomeres in the 14 chromosomes (Fig. 1b, Supplementary Data Table S3). The sizes ranged from 2177 to 126 556 bp (Supplementary Data Table S3). Tandem Repeats Finder [16] software revealed a candidate centromeric tandem repeat monomer with a length of 162 bp. We detected six putative centromeres of 14 chromosomes, with size ranging from 210 148 to 1 025 001 bp (Fig. 1b, Supplementary Data Table S4).

### Quality assessment of the telomere-to-telomere genome

Multiple methods confirmed genome completeness and continuity. Hi-C short reads data from the previous sequencing were used to validate the order and orientation of chromosomes. The signals of intra-chromosomal interaction demonstrated the satisfactory assembly of all 14 chromosomes (Supplementary Data Fig. S2). A high mapping rate of Illumina short reads (96.96%), HiFi long reads (98.72%), and ultra-long data (99.43%) aligned against the T2T genome suggest high quality of this assembly (Table 1). Additionally, we assessed the completeness of the long terminal repeat (LTR) sequences, which exhibited an LTR assembly index (LAI) value of 22.05, which meets the gold genome standard (Table 1), whereas our previously released genome had an LAI value of only 10.93 [2]. The quality assessment tool BUSCO [17] was utilized to identify the T2T genome core complete sequences. The analysis revealed that 98.5% of the core conserved genes were detected, including 1571 single-copy and 19 duplicated genes (Supplementary Data Table S5), showing improved completeness of our previously released genome (97.4%) [3]. The assembly quality of the T2T genome was also assessed using the *k*-mer method implemented in Merqury [18] based on Illumina data. The genome consensus quality value (QV) was ~53.64, with a genome completeness of 96.75% and a genome error rate of 4.32 × 10^−6^ (Table 1). Individual chromosomes displayed QV values ranging from 50.81 to 56.54, with corresponding error rates between 8.30 × 10^−6^ and 2.22 × 10^−6^ (Supplementary Data Table S6).

**Table 1:** Evaluation of *F. hispida* genome assembly quality.

**Assembly**	**T2T genome**
Number of chromosomes	14
Number of gaps	0
Assembly length (bp)	371 759 651
Illumina read-mapping rate (%)	96.96
HiFi read-mapping rate (%)	98.72
ONT read-mapping rate (%)	99.43
Genome BUSCO (%)	98.5
LTR assembly index (LAI)	22.05
Genome completeness (%)	96.75
Genome error rate	4.32 × 10^−6^
Genome QV	53.64

### Genome annotation of the T2T genome

To annotate protein-coding genes, we sequenced transcriptomes from root, stem, flower, and leaf (Supplementary Data Table S7). The initially predicted genes were filtered based on gene expression and structure. We found 26 642 protein-coding genes with an average gene coding sequence (CDS) size of 236 bp, an average of five exons per gene, and an average exon length of 238 bp, based on the *de novo*, homolog protein, and transcriptome prediction methods (Supplementary Data Table S8). BUSCO analysis [17] showed 98.7% completeness (Supplementary Data Table S6). We further identified genome-wide non-coding RNAs and obtained 513 tRNAs, 130 miRNAs, 6241 rRNAs, three sRNAs, and 577 snRNAs after screening (Supplementary Data Table S9).

We also estimated by RepeatMasker v4.10 [19] that 167.84 Mb of the assembled genome sequences is occupied by repetitive regions, accounting for 45.15% of the genome (Supplementary Data Table S10). LTR retrotransposons were the most abundant sequences, accounting for 51.5% of the repetitive sequences and 23.25% of the genome, of which Copia (3.24%) and Gypsy (15.63%) were the two top superfamilies (Supplementary Data Table S10). In addition, we identified 28.9 Mb mutator transposons, which constituted 7.8% of the whole-genome sequence (Supplementary Data Table S10).

### Improved assembly of the male-specific region

**Figure 2:**
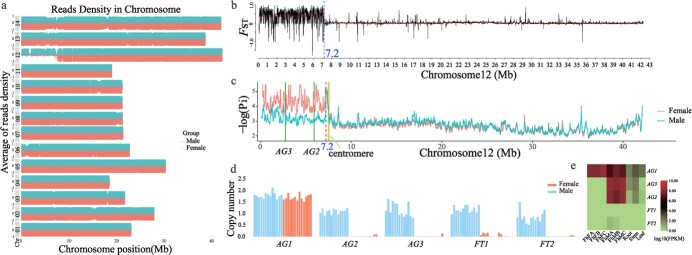
The MSR of Chr12 in the *F. hispida* genome. **a** Density distribution of second-generation resequencing reads from female and male individuals in the T2T genome. **b***F*_ST_ between female and male individuals on Chr12. **c** Comparison of nucleotide diversity (Pi) within male and female populations on Chr12. Blue dashed lines represent MSR boundaries; solid green lines are positions of sex-determining candidate genes *AG2* and *AG3*. **d** Copy number variation of *AG* and *FT* genes. **e** Expression levels of *AG* and *FT* genes in different tissues. FhFA, FhFB, and FhFC represent stages A, B, and C of female fruit development, respectively; FhMA, FhMB, and FhMC represent stages A, B, and C of male fruit development, respectively.

To investigate the male-specific region (MSR) of *F. hispida*, we re-aligned 13 male and 13 female individual resequencing reads to the T2T genome. We evaluated the read coverage in 100-kb windows for each chromosome and found an MSR near the telomere of Chr12 (Fig. 2a). We obtained 8 291 147 SNPs using our T2T genome as the reference, and calculated the *F*_ST_ values between the two sex groups based on the SNPs. Changepoint analyses detected significantly higher *F*_ST_ values between 0 and 7.2 Mb than in the rest of Chr12 (Fig. 2b). Comparative analysis of Pi (nucleotide diversity) values for females and males also detected this region (Supplementary Data Fig. S3, Fig. 2c). This MSR is ~7.2 Mb, nearly 5.2 Mb larger than previously reported [2]; 96.39% of this region is repetitive (Supplementary Data Table S11), consistent with low recombination rates.

Further annotation discovered 53 genes in the MSR (Supplementary Data Table S12). The previously reported sex-determination candidate genes *Fhv2.12G0000430* (*AG2* [2]) and the unanchored *Fhv2.12G0000210* (*AG3* [2]) were located near 5.9 and 2.7 Mb on Chr12, respectively (Fig. 2c). *AG2* and *AG3* only had a single copy in the males, while the previously reported autosomal *AG12* (*Fhv2.01G0006340*) gene had two copies in both female and male individuals (Fig. 2d). The resequencing data from 13 female and 13 male individuals detected a single copy of these genes in males but not females (Supplementary Data Table S13, Supplementary Data Fig. S4).

We next examined the expression of MSR genes across various tissues. Most MSR genes were not expressed, with a few exceptions expressed in the male flower, root, stem, and leaf (Supplementary Data Table S14, Supplementary Data Fig. S5). However, the *AG2* and *AG3* genes were highly expressed during the A and B development stages in the male flowers (Fig. 2e), and the expression level of the *AG1* gene was high in male and female flowers (Fig. 2e), as previously reported [2]. In addition to the known sex-determination candidate genes *AG2* and *AG3* previously found, we also found two FLOWERING LOCUS T-like protein genes, *Fhv2.12G0000120* (*FT1*) and *Fhv2.12G0000180* (*FT2*) in the MSR (Supplementary Data Table S12). The *FT1* and *FT2* genes are homologs of the gene *AT1G65480* in *Arabidopsis*, which plays a crucial role in floral promotion, fruit set, and vegetative growth [20]. *FT2* was slightly expressed in the A and B stages of male flower development (Fig. 2e), suggesting that it may be involved in developing early male fruits.

### Extremely high level of methylation in the male-specific region

We estimated genome-wide methylation levels based on nanopore ultra-long data. We identified 49.8, 27.9, and 2.8% methylation at CG, CHG, and CHH (H = A, T, C) sites at the whole-genome level, respectively (Supplementary Data Fig. S6). We identified 17 917 methylated genes within the genome, of which 17 740, 5642, and 3411 displayed CG, CHG, and CHH methylation, respectively. The GO functions of methylated genes were enriched in biological processes relating to ‘DNA metabolism’, ‘cell cycle’, ‘cellular component organization’, ‘regulation of gene expression’, and ‘pollen–pistil interaction’ (Supplementary Data Fig. S7).

**Figure 3:**
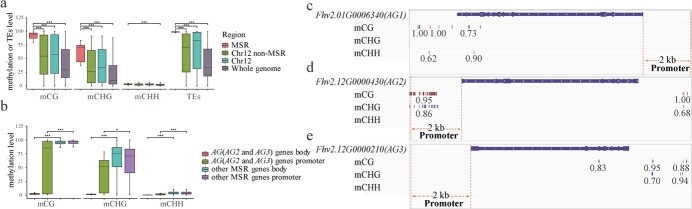
Methylation levels of MSR and genes. **a** MSR, Chr12 non-MSR, Chr12, and whole-genome mCG, mCHG, and mCHH methylation levels and transposon content comparison. **b** Comparison of mCG, mCHG, and mCHH methylation levels of *AG2* and *AG3* gene bodies, *AG2* and *AG3* gene promoter regions, other MSR gene bodies, and other MSR gene promoter regions. **c**, **d, e** mCG, mCHG, and mCHH methylation levels of gene body, promoter region, and downstream 2-kb region of different genes. The large green square represents the exon of the gene; the white arrowheads inside the blue square represent the gene transcription direction; the double orange dotted line represents the promoter region; the number represents the methylation level of this site.

To investigate the impact of methylation on the genes in the male-specific region, we first compared the MSR and non-MSR levels in Chr12, the whole Chr12 chromosome, and the whole genome. The MSR had significantly higher CG and CHG methylation levels than other genomic regions (Fig. 3a). The higher content of transposons in the ficus MSR region than in other regions may lead to the higher methylation level in the MSR (Fig. 3a), as observed in other species [21]. Gene body and promoter region methylation of the MSR region are negatively correlated with gene expression levels (Supplementary Data Fig. S8). Low expression of MSR genes may result from gene body and gene promoter hypermethylation. The gene body and promoter regions of *AG* (*AG2* and *AG3*) had significantly lower CG, CHG, and CHH methylation levels than the other MSR genes (Fig. 3b), which can explain their higher transcription levels.

We next analyzed the methylation level of the *AG* and *FT* gene bodies and their upstream and downstream 2-kb regions. CG methylation was found in exon regions of *AG1*, *AG3*, and *FT2* (Fig. 3c and e, Supplementary Data Fig. S9b), and in the introns of *AG1*, *FT1*, and *FT2* (Fig. 3c, Supplementary Data Fig. S9), while the *AG1* and *FT1* genes had CHH methylation and CHG methylation (Fig. 3c, Supplementary Data Fig. S9a), respectively. We did not detect any methylation in the promoter regions of *AG1* and *AG3* (Fig. 3c and e). However, we found a high level of methylation in the promoter region of *AG2* (Fig. 3d), which may play a role in sex regulation. Similarly, the promoter regions of the *FT1* and *FT2* genes showed high levels of methylation (Supplementary Data Fig. S9), which may affect their expression.

### Gene regulatory network differences between male and female flowers

To better characterize the transcriptional divergence between male and female flowers at different stages of development, we utilized different software to identify differentially expressed genes (DEGs) between male and female flowers. We identified 1250, 839, and 4326 DEGs in stages A, B, and C of female and male flower development, respectively (Supplementary Data Fig. S10). Next, we constructed a gene regulatory network for male and female flower development genes. We observed that network memberships and size differences between males and females at different stages of development increase with fig flower development for different sexes (Supplementary Data Table S15). We compared the topology of the gene regulatory networks between males and females at different developmental stages. The female network had a higher average number of neighbors, characteristic path length, clustering coefficient, and number of edges than the male network during the A and C stages of flower development, indicating that the female gene regulatory network was more robust and complex (Supplementary Data Table S15). However, the male gene regulatory network had a higher numbers of nodes, edges, and neighbors and a higher clustering coefficient in the B stage than the female network (Supplementary Data Table S15), suggesting a more complex network than that of the female.

**Figure 4:**
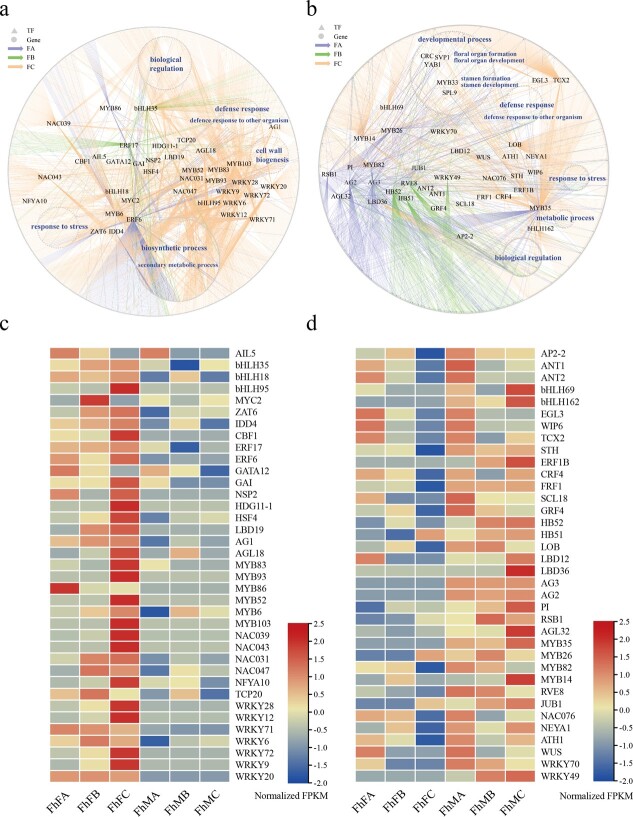
Gene regulatory networks of the different sexes in ficus. **a** Female gene regulatory network of ficus. Gray triangles represent transcription factors; gray circles represent other genes (except for TF); the purple arrow (FA) represents fruit development stage A; the green arrow (FB) represents fruit development stage B; and the orange arrow (FC) represents fruit development stage C. **b** Male gene regulatory network of ficus. **c** Expression levels of transcription factors in the female gene regulatory network at different fruit development stages. **d** Transcription factor expression as different fruit development stages in the male gene regulatory networks.

We compared the functional differences between female and male network members’ development. The C developmental stage network was the most complex in the male and female gene regulatory network compared with other stages (Fig. 4). The gene function enrichment analysis in the female and male networks showed that they shared the same critical biological processes, such as ‘natural regulation’, ‘response to stress’, ‘metabolic process’, and ‘defense response’ (Supplementary Data Tables S16and S17, Fig. 4). However, ‘cell wall biogenesis process’ was explicitly enriched in the female gene regulatory networks (Supplementary Data Table S16, Fig. 4a). The male network was enriched explicitly for the development process, including ‘floral organ formation and development’ and ‘stamen formation and development’ (Supplementary Data Table S17, Fig. 4b). For flower organ formation and development, the upregulated genes in male fruit included *CRABS CLAW protein* (*CRC*), *SQUAMOSA PROMOTER BINDING PROTEIN-LIKE 9 protein* (*SPL9*), *MYB DOMAIN PROTEIN 33 protein* (*MYB33*), *SHORT VEGETATIVE PHASE* (*SVP1*), ethylene-responsive element binding protein (*AP2*), MADS domain transcription factors (*AG2*, *PI*, and *AG3*), and a member of the YABBY family of transcriptional regulators (*YAB1*) (Fig. 4b). Statistical analysis of the transcriptional regulators of the gene regulatory network showed that 37 and 36 transcription factors were upregulated in the female and male, respectively (Fig. 4). The prominent transcription factor families involved in the female gene regulatory network were MYB, NAC, and WRKY. In contrast, the leading families in the male gene regulatory network were MADS and MYB transcription factors (Supplementary Data Fig. S11).

## Discussion

A previous study has identified a sex-determination region that spans ~2 Mb on Chr12 of *F. hispida.* A sex-candidate gene, *FhAG2*, a member of the MADS-box transcription factor family, was discovered within this region. Two additional homologous genes, *FhAG1* and *FhAG3*, were found in other genome regions, with *FhAG1* located on Chr01 and *FhAG3* on a contig [2]. However, we identified a larger MSR at ~7.2 Mb, with more genes than previously. The gene *AG2*, a candidate sex-determination gene, was located at near the 5.9 Mb position in the new MSR. Further, we have re-anchored the new sex-determination gene *AG3* to the newly assembled MSR, specifically near the 2.7 Mb position. Two male-specific *FT*s (*FT1* and *FT2*) were first identified in the MSR, of which *FT2* was explicitly expressed in the A and B stages of male flowers. Many studies have demonstrated the vital role of the *FT* gene in flowering, fruit set, and vegetative growth, and the positive regulation of flower development [22–25]. It is suggested that male-specific *FT* genes may be related to the growth of male flowers in *Ficus*. Additionally, methylation analysis of the MSR region suggested that methylation may play an important role in shaping the expression and function of MSR genes.

The gene regulatory network is a valuable tool for understanding the interactions between genes on a large scale. It can predict regulatory relationships and key biological pathways within the network [26]. Comparative analysis of the female and male gene regulatory networks revealed that distinct regulatory mechanisms may be involved in developing male and female flowers. The symbiosis between the ficus and wasp has existed for ~75 million years of coevolutionary history [7], and ficus–wasp symbiosis selection has also become an essential driving force for the synergistic diversification and trait evolution of both species [27]. During the flower development of female plants, the female flower will produce some volatile substances to attract pollinators to enter the ficus fruit for pollination. We identified biological pathways related to secondary metabolites in the female gene regulatory network, such as the aromatic compound biosynthetic process. The cell wall biogenesis pathway was enriched at the C stage in the female gene regulatory network, indicating that the female fruit’s cell wall was developing and the fruit was expanding. Furthermore, *AG1* gene regulation is involved in defense responses to deter infestation by non-pollinating wasps and insects in the female gene regulatory network. In the male gene regulatory network, genes *AG2* and *AG3* interact in floral organ formation/development and stamen formation/development. We identified several genes related to flower development that *AG2* and *AG3* regulated during the formation and development of male flowers. Our study provides valuable insights into the regulatory mechanisms underlying male and female flower development in ficus and the role of *AG* (*AG1, AG2*, and *AG3*) genes in ficus–wasp mutualism.

## Materials and methods

### Genome sequencing, assembly, and quality assessment

The ficus plants for genome sequencing were cultivated in Xishuangbanna Tropical Botanical Garden, Chinese Academy of Sciences in Menglun Town, Mengla County, Xishuangbanna Dai Autonomous Prefecture, Yunnan Province of China. We collected healthy leaves and immediately froze them in liquid nitrogen to extract DNA. After extracting DNA by the modified cetyl-trimethylammonium bromide (CTAB) method [28], RNase A (Invitrogen) was used to eliminate RNA contamination and DNA quality was evaluated by electrophoresis on 1.5% agarose gels. For PacBio sequencing, a 15-kb SMRTbell library was constructed from genomic DNA according to the manufacturer’s instructions. This library was sequenced on the PacBio Sequel II platform. The adapters and low-quality reads were removed to obtain high-quality and clean subreads. For nanopore ultra-long DNA sequencing, an extra-long reads library was built from high-quality DNA, which was fragmented to 100 kb according to the library building manual. The high-quality reads with *Q*-score ≥7 were reserved for further analysis.

The consensus reads were obtained using CCS software (https://github.com/PacificBiosciences/ccs) with the default parameter. The long (~15 kb) and highly accurate (99%) HiFi reads were initially assembled using hifiasm [29] software with default parameters to generate a primary contig genome as the backbone of the genome. We used Hi-C data to anchor and remove low-quality short contigs to improve genome assembly quality further. The clean reads were mapped to the assembled genome by bwa-0.7.17 [30] with the mem model. The ALLHiC [31] algorithm was used to correct, cluster, and orient the contigs. The genome generated with HiFi and ONT data formed 14 large contigs representing 14 chromosomes. We further error-corrected the genome by Juicebox [32] software according to the interaction signal. Finally, HiCExplorer [33] software was used to plot the heat map of genome interactions.

The ficus genome completeness was assessed by BUSCO [17] with the single-copy genes embryophyte_odb10 database. The genome accuracy was evaluated by mapping the whole-genome sequencing data to the genome using bwa-0.7.17 with the mem model and calculating the mapping rate and coverages with Qualimap2 genome sequencing, assembly, and quality assessment [34]. The continuity of the genome was estimated by calculating the contig N50 length. We also assessed the assembled genome using the LAI value [35].

### Genome annotation

Transposable elements and tandem repeats were annotated based on the *de novo* and homology-based methods. First, a *de novo* repeat library was constructed using the RepeatModeler [36] program with the default parameters. Then, the intact retrotransposons were detected using both LTRharvest [37] and LTR_finder [38]. We used LTR_retriever [39] software to build a high-quality and non-redundant intact LTR library. A non-redundant specific-specific transposable element library was constructed by merging the known Rephase v19.06 and REXdb v3.0 database with the sequence library above. Finally, the genome transposable element sequences were determined and classified by homology searches against the library using RepearMasker v4.10 [40]. The Tandem Repeats Finder identification tool [16] with default parameters was used to annotate tandem repeats.

Protein-coding gene prediction and annotation were integrated using *ab initio* gene prediction, homology-based gene search prediction, and RNA-seq assembly prediction. The ficus genome repeats were masked by RepeatMasker [40] software before gene structure prediction. The *de novo* gene prediction software Augustus v2.4 [41] and SNAP [42] were used to perform *ab initio* gene prediction. For the homology-based approach, the homology protein sequences from the model plants or related species *Arabidopsis*, *Morus alba*, *Broussonetia papyrifera*, *Ficus carica*, *Ficus macrocarpa*, and *Ficus hispida* were aligned to the T2T genome, and the protein coding genes were predicted using Exonerate v2.2.0 (https://github.com/nathanweeks/exonerate) software. The RNA-seq reads were mapped to the reference genome using HISAT2 [43, 44] with the default parameters and assembled by StringTie [45]. The PASA [46] software tool was used to predict gene characteristics based on the assembled transcripts, and the complete gene structure was selected for training using Augustus v2.4 [41]. The final gene models from prediction were integrated by EVidenceModeler [47] software.

Non-coding RNAs were also annotated in the genome, including tRNAs, rRNAs, miRNAs, snoRNAs, and snRNAs. The program tRNAscan-SE [48] was used to identify tRNA genes with particular criteria for eukaryotes and default parameters. miRNA sequences were identified using miRbase [49] with default parameters. RNAmmer [50] was used to predict rRNAs genes, and snoRNA and snRNA genes were annotated by an Infernal [51] search of the Rfam [52] database.

### Telomere and centromere detection

The plant telomere sequences (CCCTAAA) of each genome chromosome were searched, and 27 of the 28 telomeres were detected. For telomere-missing chromosomes, telomere reads were extracted from HiFi and ONT reads, and the reassembled telomere sequence was patched to the telomere-missing chromosomes. Tandem Repeats Finder [16] was used to identify candidate repeat centromere sequences. The approximate boundary of the centrosome region was estimated from the frequency of all candidate repeat sequences.

### DNA methylation prediction and analysis

The frequency of genome-wide CpG methylation was detected using raw Oxford Nanopore Ultra-long data based on the DeepSignal-plant [53] standardized workflow. First, a new model for detecting 5mC methylation was trained using ficus nanopore reads according to the DeepSignal-plant [53] flow. Then, the ficus raw fast5 files were base-called and re-squiggled by Guppy v3.6.1 and Tombo v1.5.1, respectively. Finally, the deep-learning algorithm called CG-, CHG-, and CHH-type methylation sites. The C-methylated site with read coverage of less than five was removed to exclude false positives.

The methylation level of a specific C-site (CG, CHG, and CHH) was determined by calculating the ratio of reads supporting methylation modifications to the total number of reads mapped to that C-site. If this ratio exceeds 0.5, the C-site is considered methylated [53]. Moreover, when CG, CHG, or CHH methylation occurs in a gene’s CDS region, the gene is categorized as a methylated gene. The methylation level of a region is equal to the ratio of the number of reads supporting a methylation modification at the C site to the number of all reads aligning the region.

The average methylation of genes and transposon regions was calculated using ViewBS [54] software based on the genome-wide methylation frequency files identified by the DeepSignal-plant [53] software. The MethOverRegion model in the ViewBS [54] software was used to estimate the methylation level of the gene and transposon regions and its upstream and downstream 2 kb, with the parameters: —binNumber 20 —flank 2000 —binLength 200 —minDepth 5 —maxDepth 400. The ggplot2 package in the R language was used for visualization.

### Transcriptome analysis

The RNA sequencing data used in this study were obtained from a published paper (Supplementary Data Table S5) [2]. The ficus female and male fruit samples from three developmental periods and leaf and stem samples were used for transcriptome analysis, and three biological replicates were contained in each sample. The adapters and low-quality bases were removed from RNA-seq raw data using Trimmomatic v0.33 [55] with the parameters PE -phred64 ILLUMINACLIP: TruSeq3-PE. fa:2:30:10 LEADING:3 TRAILING:10 SLIDINGWINDOW:3:15 MINLEN:100. The high-quality clean RNA reads were aligned to the ficus T2T reference genome using Bowtie2 [56] with default parameters. FPKM (fragments per kilobase pair per million reads) values were counted using RSEM [57] software. We used different methods, DESeq2 [58] and edgeR [59] , to identify DEGs in different tissues and developmental periods. We set fold change ≥2 and false discovery rate ≤0.01 as the DEG criterion between sample and sample, and the DEGs were further used to construct gene regulatory networks.

### Gene regulatory network construction

Dynamic changes in gene expression regulation during different developmental periods between males and females in ficus flowers (fruits) were investigated. We used the mutual ranking (MR) algorithm [60] to identify the hierarchical co-expression relationships between genes and genes. Pearson correlation coefficient (PCC) analysis was used to determine the correlation between two genes from the DEGs. A correlation coefficient value between two genes ≥0.9 was considered positive, and a value ≤−0.9 was a negative correlation. The correlation coefficient values of these genes with positive or negative correlations were used as input data to calculate MR values further. The geometric mean between the PCC rank values of gene 1 to gene 2 and gene 2 to gene 1 was the MR value; thus, MR values range from 0 to 1, with 1 representing significant interaction between gene 1 and gene 2. All interacting gene pairs with MR ≥0.90 were used to construct the gene co-expression network. To further investigate the regulatory relationships between interacting gene pairs, we used PlantPAN [61] to identify 2349 encoding transcription factor genes for downstream analysis in the ficus genome. The 2-kb upstream sequence of the gene start site was used as the gene’s promoter sequence in the ficus genome to scan for *cis*-regulatory elements (CREs). The known transcription factor binding site databases JASPAR Plantae (http://jaspar.genereg.net/) and PlantTFDB (http://planttfdb.gao-lab.org/) were used to predict CREs in the promoter regions of target genes. The AME tool was used to count the overrepresentation of CREs from DEG promoter sequences and the overexpression of specific transcription factor families. The motif enrichment method for promoter regions was used with Fisher’s exact test, and the *P*-value <0.001 was considered to indicate a candidate for meaningful enrichment results. The corresponding gene was retained in the regulatory network. Finally, the gene regulatory network contained co-expression (interaction) relationships between pairs of DEGs and predicted differentially expressed transcription factor regulation of target genes. We applied Cytoscape [62] v3.6.1 for further statistical analysis and visualization of the gene regulatory network.

## Acknowledgements

This work was supported by the National Natural Science Foundation of China (grant 32200188) and the Key Programs of Jiangxi Youth Science Foundation (grant 20202ACBL215008).

## Author contributions

X.T.Z. and G.W. designed and guided the project; Z.Y.L., Y.B.W., J.Y., and K.C. performed genome assembly and gene annotation; Z.Y.L. and W.L.L. performed methylation analysis; Z.Y.L. and T.W.Z. built gene regulation networks; T.W.Z and G.W. collected the samples; Z.Y.L. conducted other analyses; Y.H.W. helped to revise the article figures and tables; Z.Y.L. wrote the manuscript; Z.Y.L. and X.T.Z. revised the paper; H.H.Z. assisted in polishing the language of the article. All authors read and approved the manuscript.

## Data availability

The genome sequences described in this article have been submitted to The National Genomics Data Center (NGDC, https://ngdc.cncb.ac.cn) under accession number PRJCA016767 (whole genome and assembly data).

## Conflict of interests statement

The authors declare that they have no conflict of interest.

## Supplementary Data

Supplementary data is available at *Horticulture Research* online.

## Supplementary Material

Web_Material_uhad257
