# Supplementary material for: A telomere-to-telomere reference genome of ficus (Ficus hispida) provides new insights into sex determination
Source: Hortic Res. 2023 Dec 13;11(1):uhad257. doi: 10.1093/hr/uhad257 (PMC10807705; doi:10.1093/hr/uhad257)
Supplement: Web_Material_uhad257 [file web_material_uhad257.zip › Supplementary Figure.pdf]

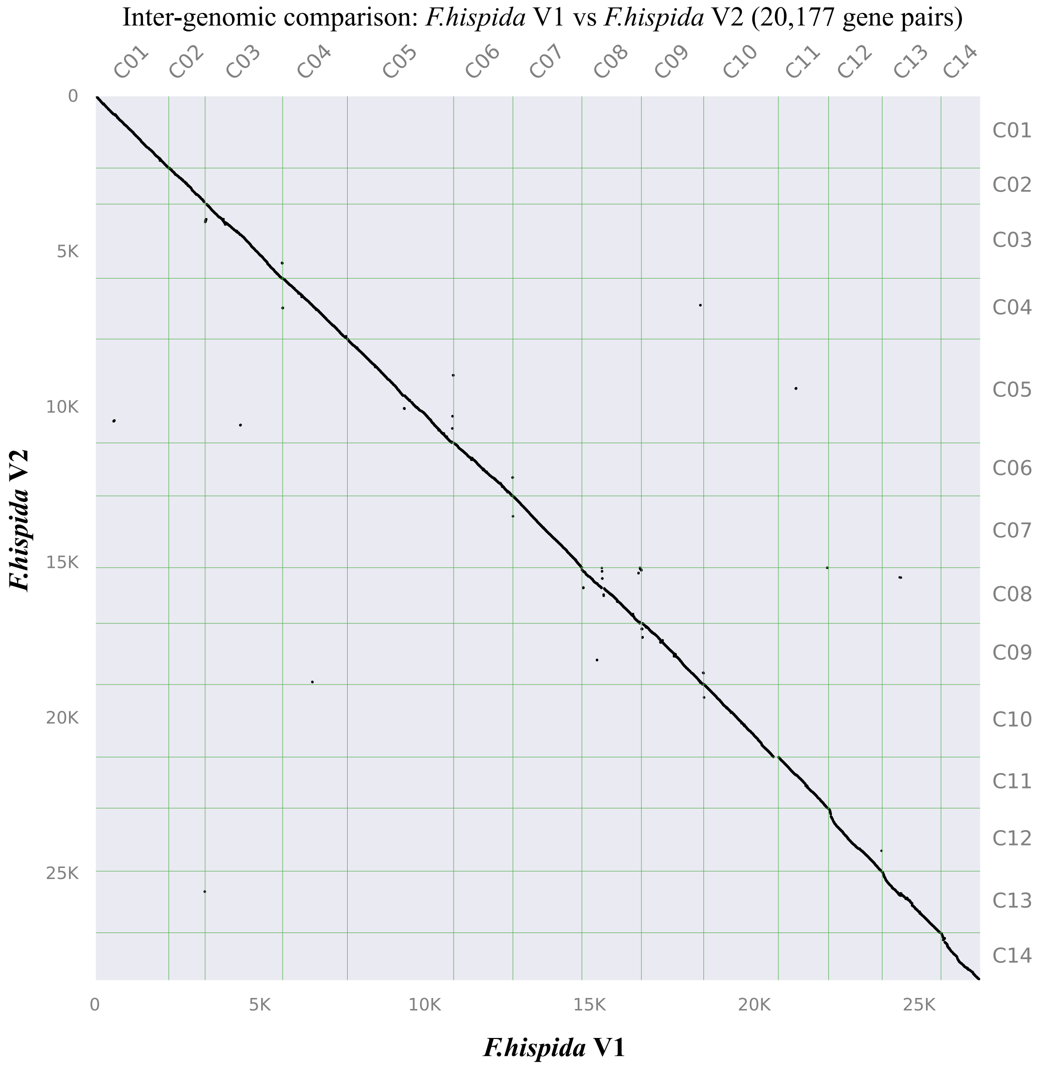

Supplementary Figure 1 The gene collinearity analysis for different versions of*F.hispida* genome, the V2 version is the near-complete genome.

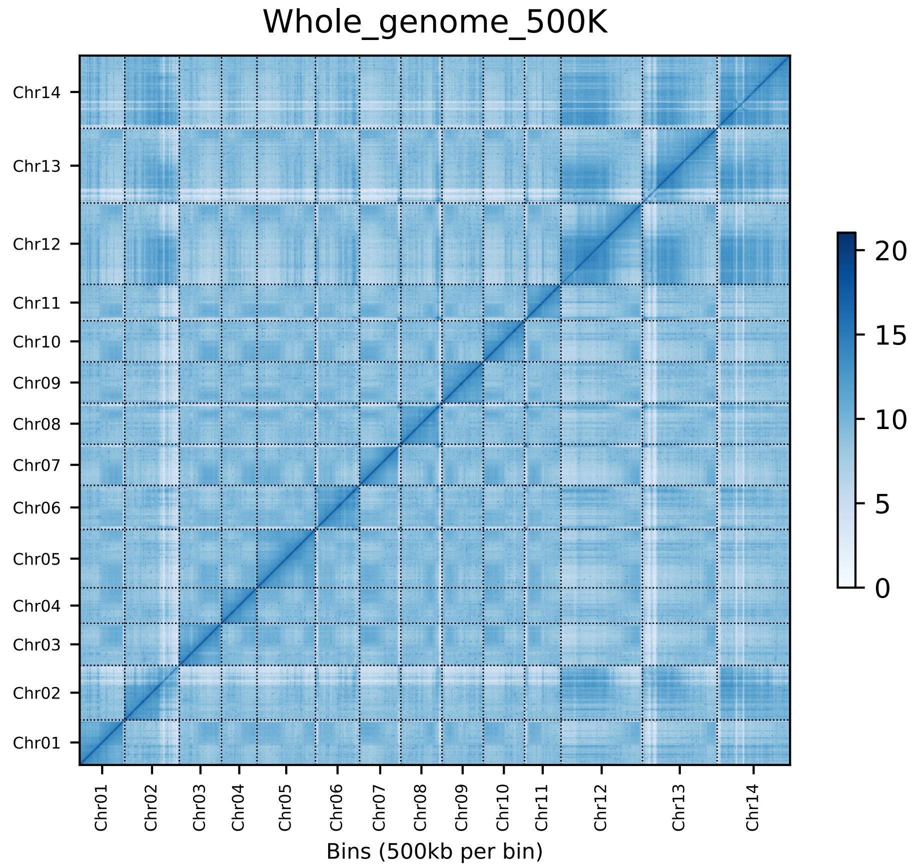

Supplementary Figure 2 Intensity signal heat map of the high-throughput chromatin conformation capture (Hi-C) chromosome interaction.

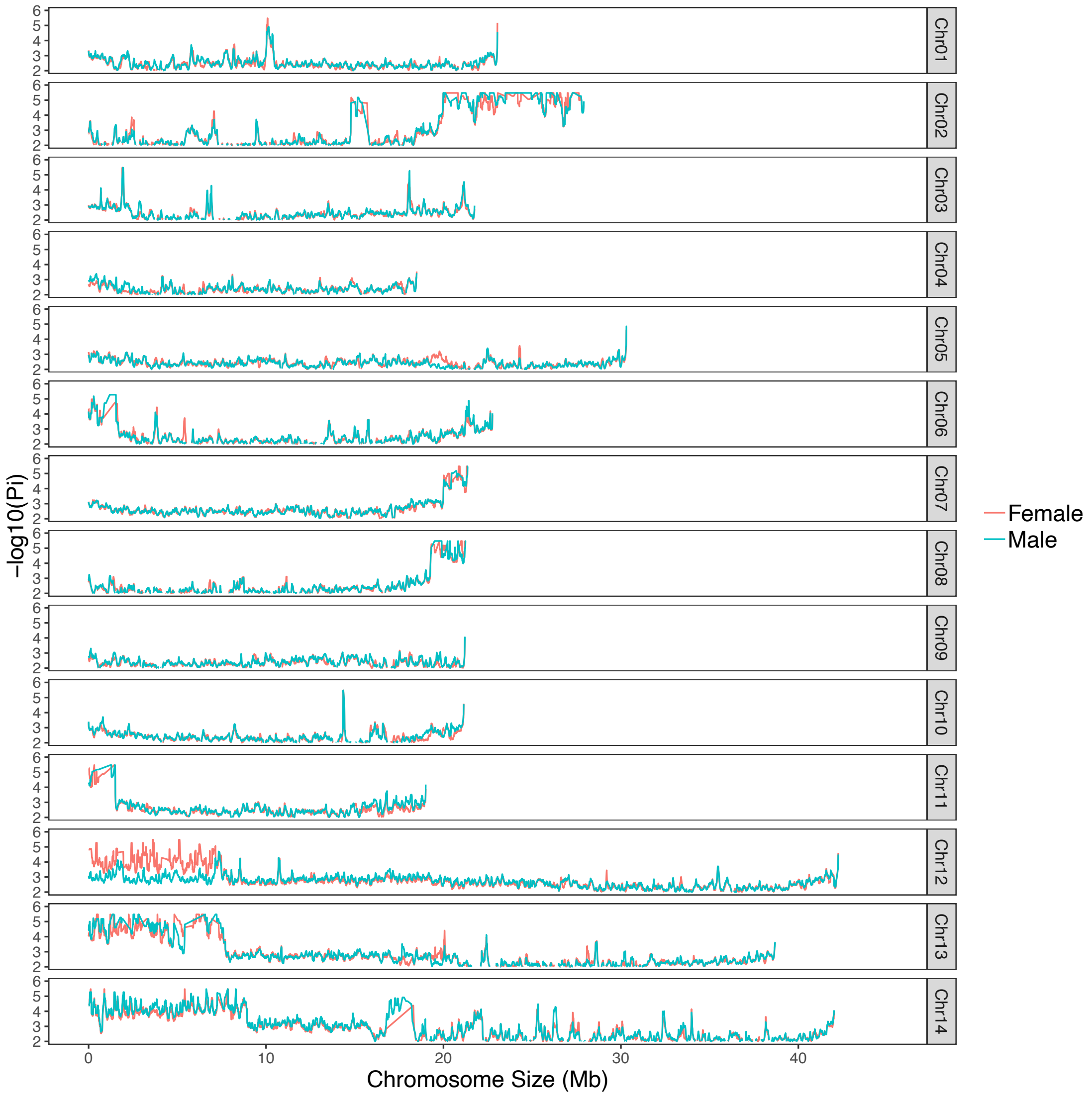

Supplementary Figure 3 The genetic diversity analysis of different sexes of each chromosome was based on the second-generation resequencing data using the sliding window method (window 100kb, step size 10kb).

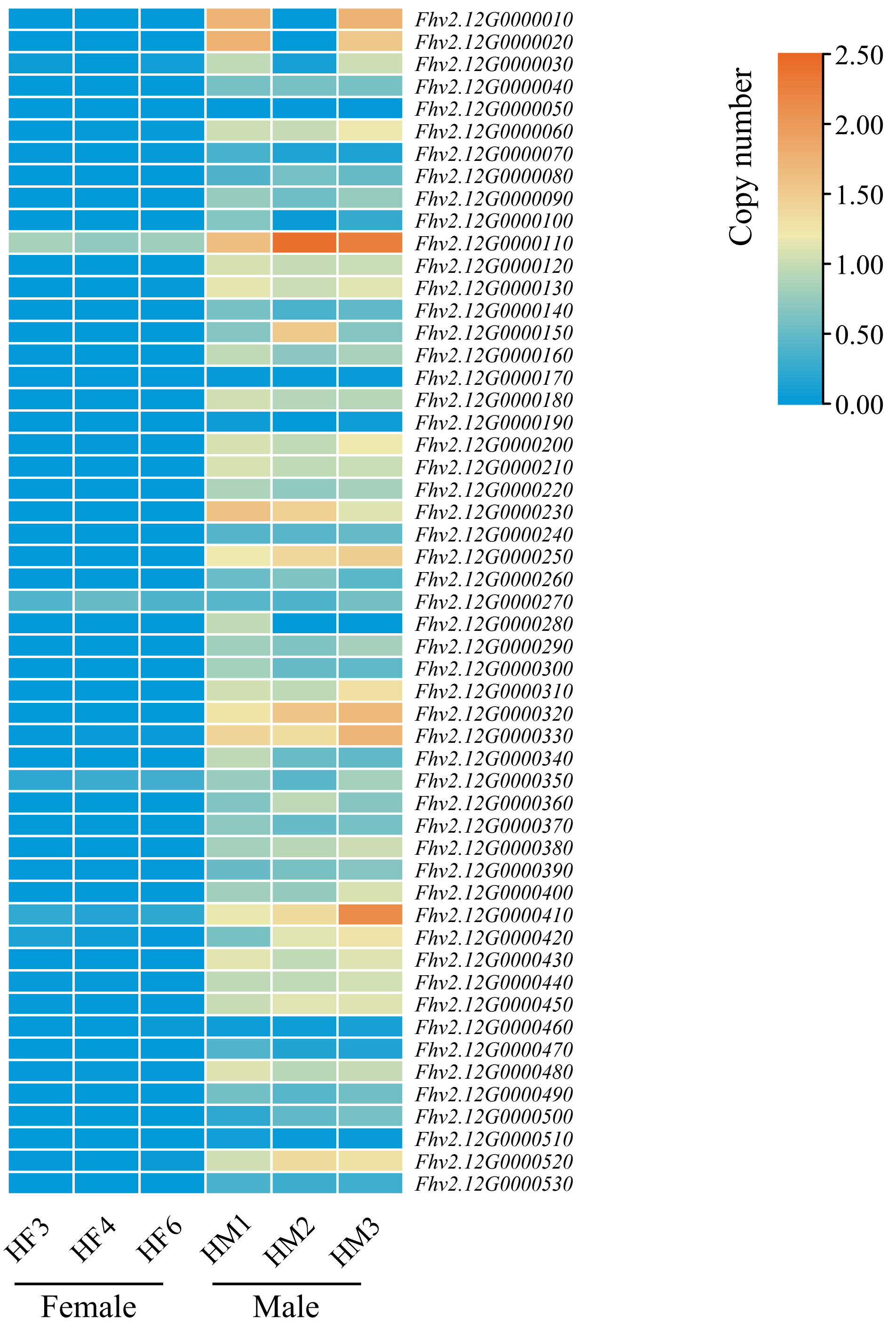

Supplementary Figure 4 Heat map of gene copy number variation of SDR genes; HF3, HF4, and HF6 represent three female plants; HM1, HM2, and HM3 represent three male plants.

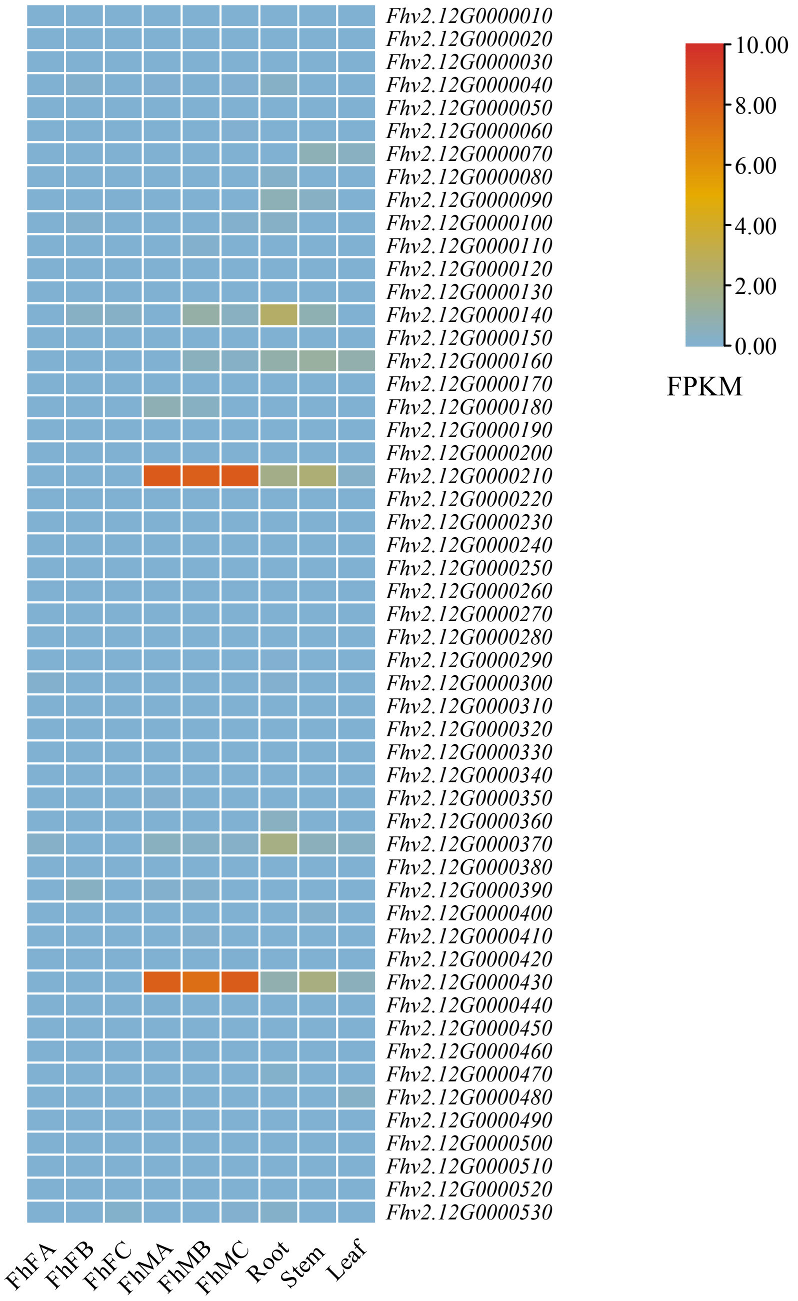

Supplementary Figure 5 The expression levels of SDR genes in different tissues; FhFA, FhFB, and FhFC represent stages A, B, and C of female fruit development, respectively; FhMA, FhMB, and FhMC represent stages A, B, and C of male and female fruit development, respectively. FPKM: Fragments Per Kilobase of exon model per Million mapped fragments.

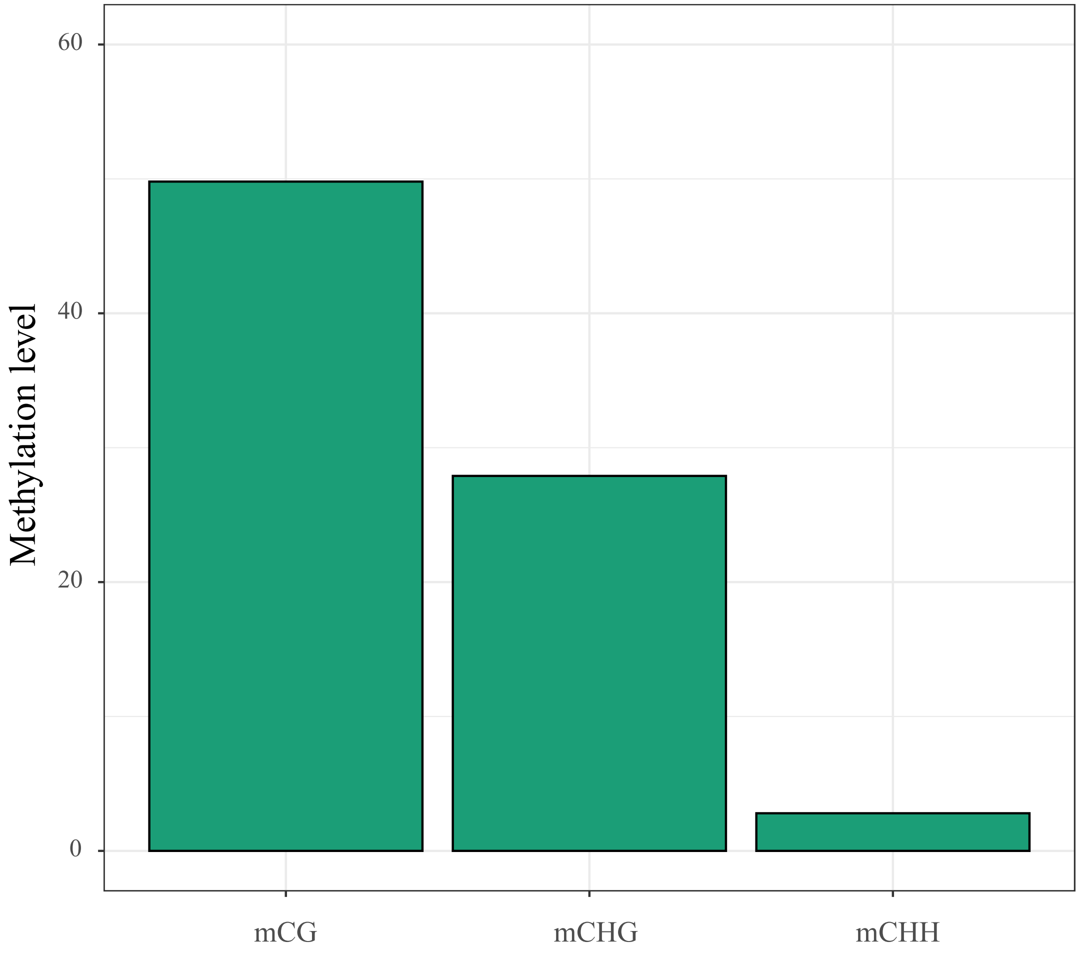

Supplementary Figure 6 Genome-wide average mCG-type,mCHG-type, and mCHH-type methylation level.

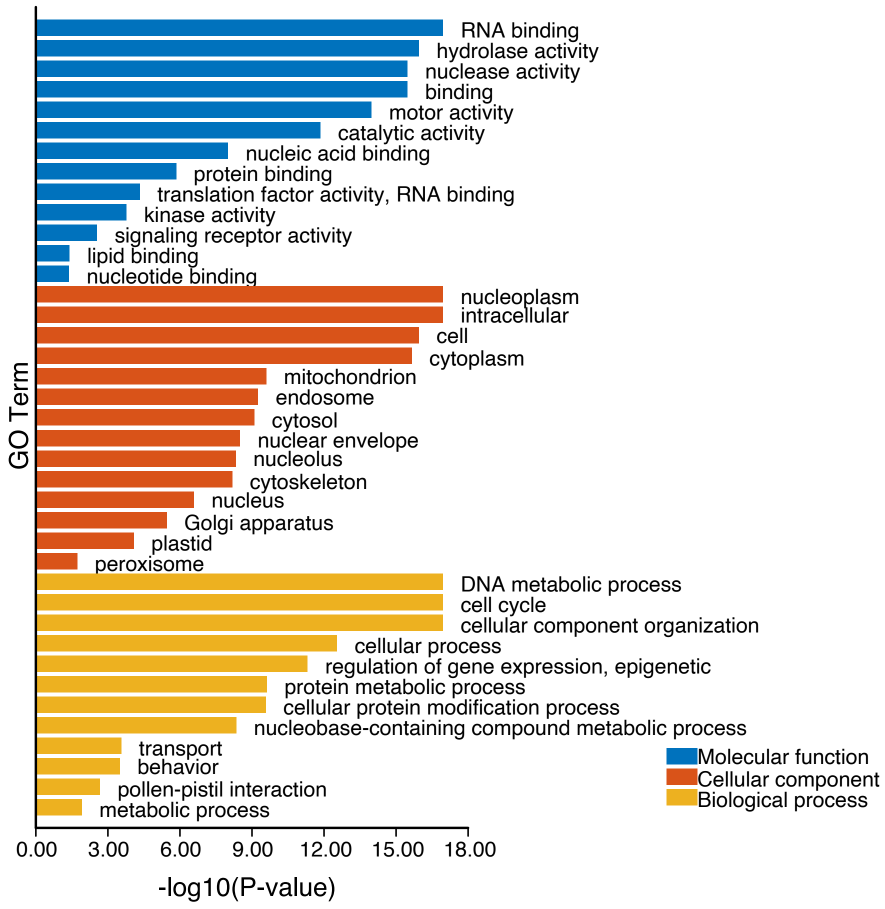

Supplementary Figure 7 GO functional enrichment analysis of methylated genes

a

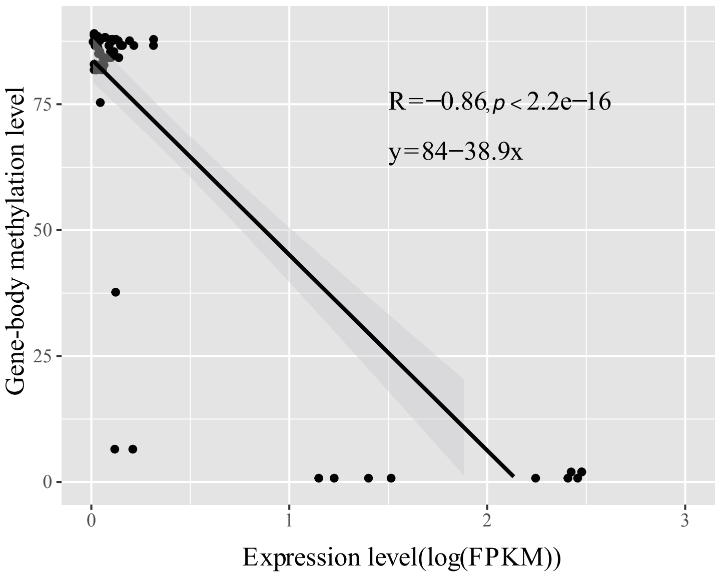

b

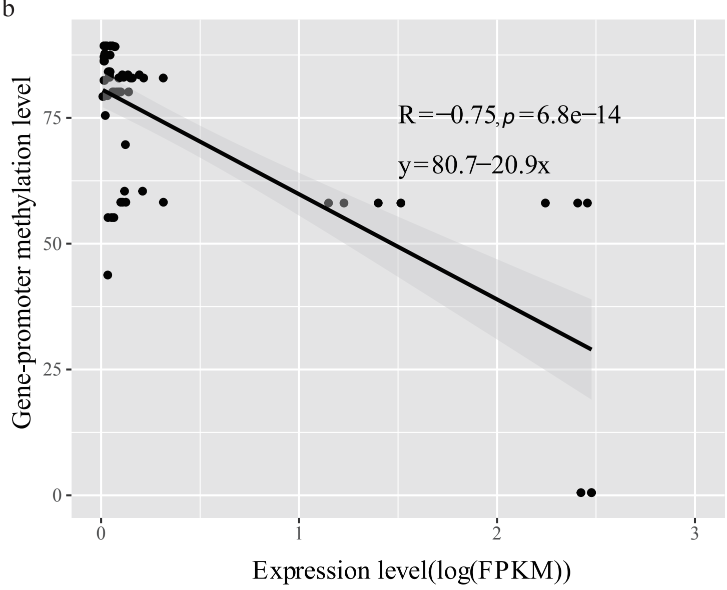

Supplementary Figure 8 Relationship between methylation and gene expression levels. a: Correlation between gene expression levels and gene-body methylation in the SDR region. b: Correlation between gene-promoter methylation in the SDR region and gene expression.

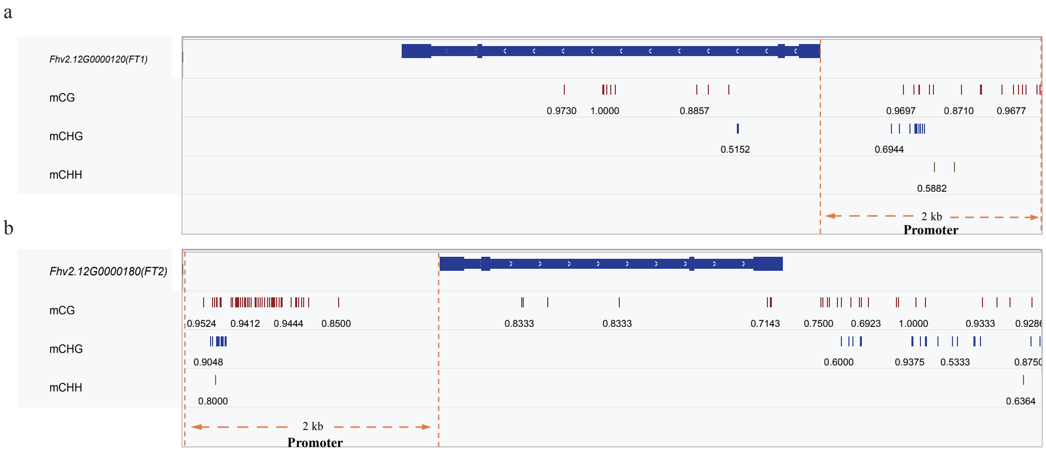

Supplementary Figure 9 The mCG-type, mCHG-type, and mCHH-type methylation levels of gene body, promoter region, downstream 2kb region of different genes. a: *FT1*; b: *FT2*.

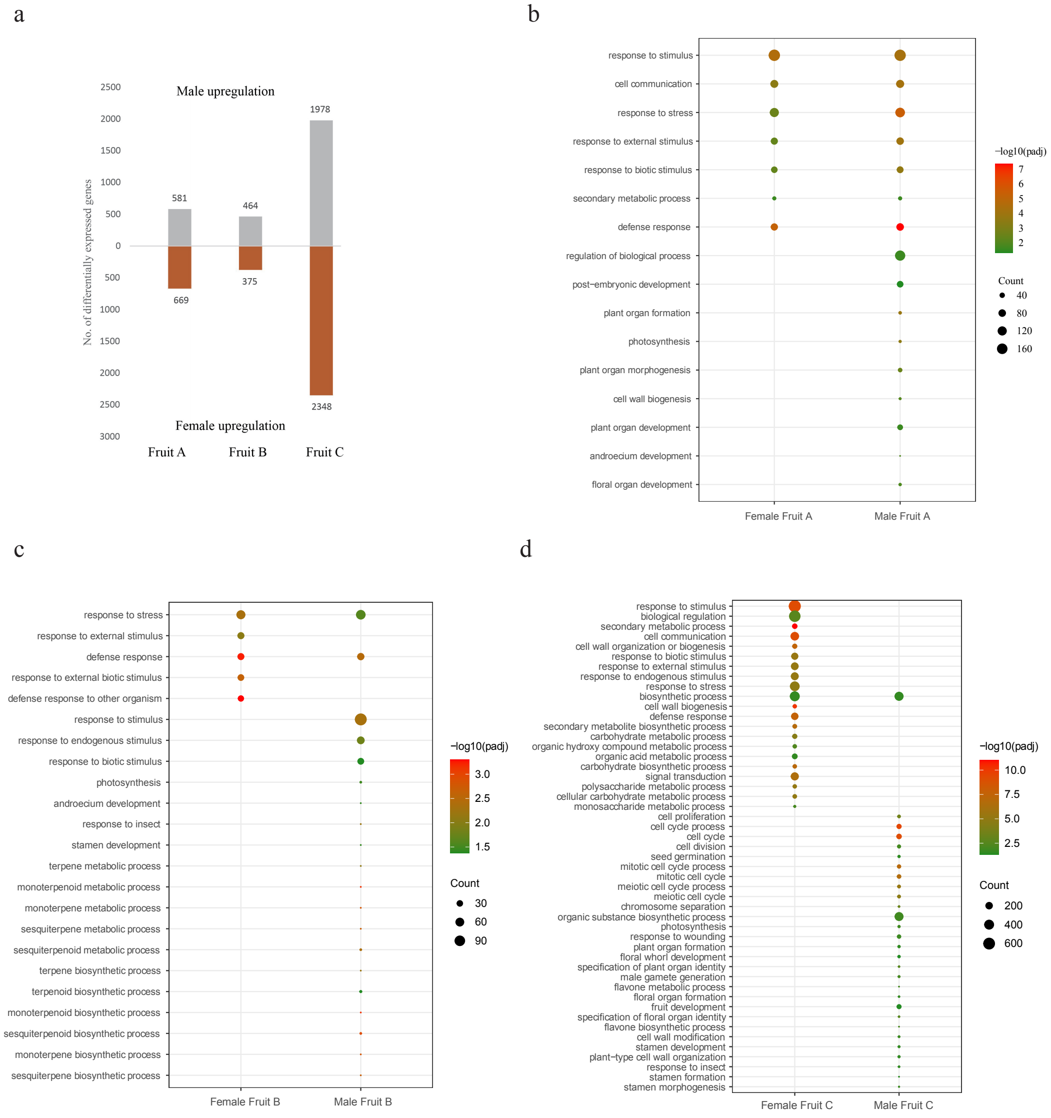

Supplementary Figure 10 Transcript analysis at different developmental stages of fruit. a: The number of genes differentially expressed during fruit A, B, and C development between males and females. b: GO enrichment analysis of up-regulated genes in females and up-regulated genes in males during fruit development stage A. c: GO enrichment analysis of up-regulated genes in females and up-regulated genes in males at stage B of fruit development. d: GO enrichment analysis of upregulation genes of females and upregulation genes of males at stage C of fruit development.

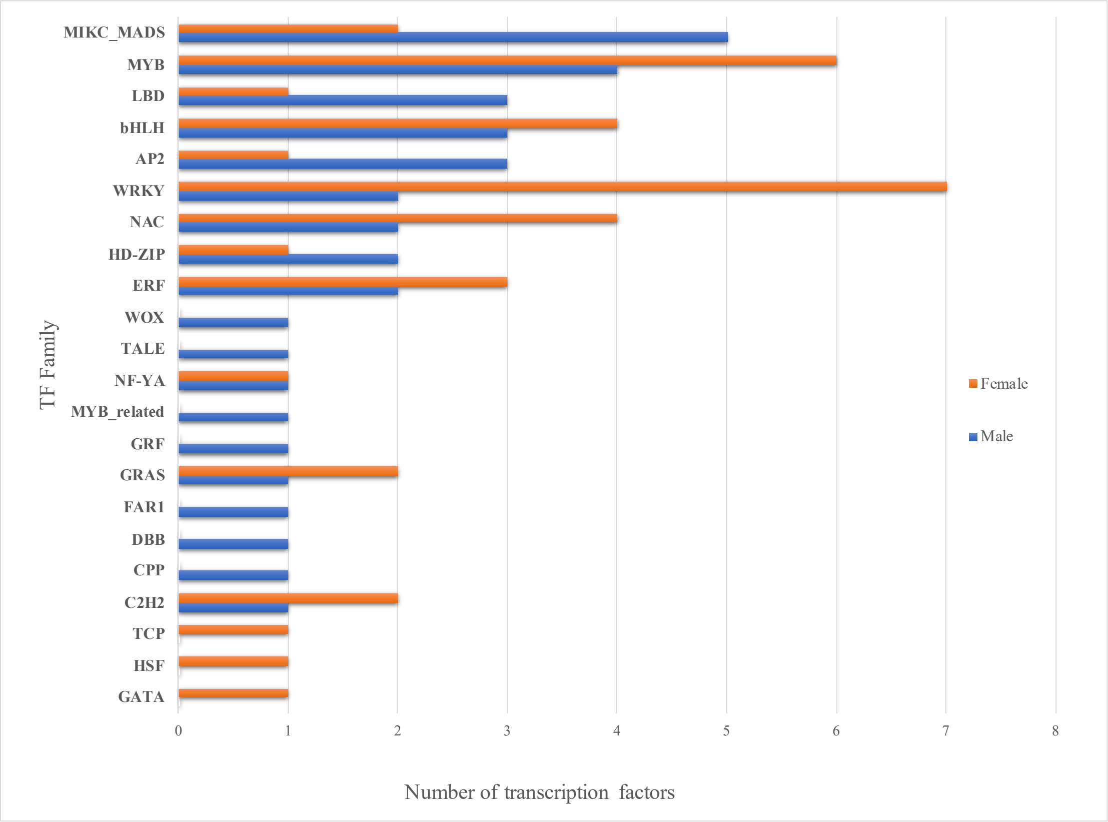

Supplementary Figure 11 Counting the number of transcription factor families in the gene regulatory network of female fruit and male fruit.
